# Could future ocean acidification be affecting the energy budgets of marine fish?

**DOI:** 10.1093/conphys/coae069

**Published:** 2024-10-08

**Authors:** Gwangseok R Yoon, Arsheen Bozai, Cosima S Porteus

**Affiliations:** Department of Biological Sciences, University of Toronto Scarborough, 1265 Military Trail, Toronto, Ontario, M1C 1A4, Canada; School of Marine and Environmental Programs, University of New England, 11 Hills Beach Road, Biddeford, Maine, 04005, USA; Department of Biological Sciences, University of Toronto Scarborough, 1265 Military Trail, Toronto, Ontario, M1C 1A4, Canada; Department of Biological Sciences, University of Toronto Scarborough, 1265 Military Trail, Toronto, Ontario, M1C 1A4, Canada

**Keywords:** Ocean acidification, metabolic rate, locomotion, digestion, growth, reproduction, fish

## Abstract

With the unprecedented environmental changes caused by climate change including ocean acidification, it has become crucial to understand the responses and adaptive capacity of fish to better predict directional changes in the ecological landscape of the future. We conducted a systematic literature review to examine if simulated ocean acidification (sOA) could influence growth and reproduction in fish within the dynamic energy budget theory framework. As such, we chose to examine metabolic rate, locomotion, food assimilation and growth in early life stages (i.e. larvae and juvenile) and adults. Our goal was to evaluate if acclimatization to sOA has any directional changes in these traits and to explore potential implications for energetic trade-offs in these for growth and reproduction. We found that sOA had negligible effects on energetic expenditure for maintenance and aerobic metabolism due to the robust physiological capacity regulating acid–base and ion perturbations but substantive effects on locomotion, food assimilation and growth. We demonstrated evidence that sOA significantly reduced growth performance of fish in early life stages, which may have resulted from reduced food intake and digestion efficiency. Also, our results showed that sOA may enhance reproduction with increased numbers of offspring although this may come at the cost of altered reproductive behaviours or offspring fitness. While these results indicate evidence for changes in energy budgets because of physiological acclimatization to sOA, the heterogeneity of results in the literature suggests that physiological and neural mechanisms need to be clearly elucidated in future studies. Lastly, most studies on sOA have been conducted on early life stages, which necessitates that more studies should be conducted on adults to understand reproductive success and thus better predict cohort and population dynamics under ongoing climate change.

## Introduction

Fish are the most diverse vertebrate group with over 34 000 species and have a wide distribution on our planet. Fish are central to various ecological processes such as energy and nutrient transfer between niches in the food web (Barneche and Allen, 2018), mixing water columns through physical activity (Lauder, 2015) and ecological memory as an adaptive evolutionary response to their environment (Hughes *et al*., 2019). As the environment could strongly influence behaviour and physiology of fish, it is crucial to understand their responses and adaptive capacity under the ongoing rapid climate change to better predict directional changes in the ecological landscape of the future (Gordon *et al*., 2018).

In the past century, we have witnessed a dramatic increase in atmospheric carbon dioxide (CO_2_) mostly derived from anthropogenic activities (Stocker *et al*., 2013). Over a quarter of this in turn has been absorbed by the ocean, causing ocean acidification (OA). Under the business-as-usual model, it is predicted that CO_2_ in the open ocean will increase from the current level of 400 μatm–1000 μatm while pH will decrease by 0.3 unit (Stocker *et al.,* 2013). Although these physical changes seem to be negligible, it has been predicted that OA may exacerbate CO_2_ fluctuations in the coastal environments in which aquatic organisms may already live near their physiological limits (Baumann, 2019; Munday *et al*., 2019). Although our understanding of behavioural and physiological effects of OA on fishes has been remarkably improved in the past decade (Jutfelt *et al*., 2013; Baumann, 2019; Munday *et al*., 2019), what has remained neglected is our lack of a biological framework that theorizes potential mechanisms to explain various processes involved in energy metabolism that could ultimately influence growth and reproduction under OA (Sousa *et al*., 2010).

The dynamic energy budget (DEB) theory is a biological framework that explains the compartmentalization of energy metabolism such as energy intake, assimilation and allocation to support biological processes at the individual level (Koojiman, 2009). DEB takes an integrated approach of thermodynamics, biophysics and physiology to provide a mechanistic insight for understanding various processes involved in metabolism such as maintenance, locomotion, digestion, growth and reproduction (Sousa *et al*., 2008). DEB has been widely used in ecology to understand the metabolic impacts that environmental changes could have on individuals and thus predict population dynamics (Lavaud *et al*., 2021).

**Figure 1:**
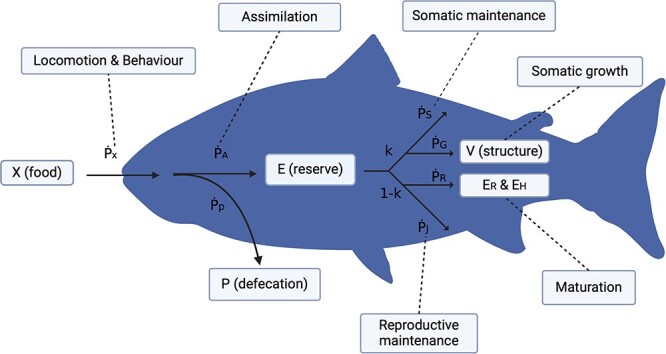
Conceptual diagram of the dynamic energy budget in a marine fish. Food (X) in the form of chemical energy is ingested by the fish (${\dot{P}}_X$), which is further mobilized into the energy flux of assimilation (${\dot{P}}_A$) and defecation (${\dot{P}}_P$), giving rise to the amount of energy in the reserve (E) and defecation (P). The amount of energy in the reserve (E) is then used towards somatic allocation (k) and anything else (1 − k; e.g. reproduction), both of which are assumed to be energetically independent in the model. The energy flux of somatic category (k) is comprised of somatic maintenance (${\dot{P}}_S$) and somatic growth (${\dot{P}}_G$), with the balance energy allocated to structure (V). The reproduction (1 − k) has the similar scheme for reproductive maintenance (${\dot{P}}_R$) and reproductive reserve (${\dot{P}}_H$), giving rise to the reproductive development (${E}_H$) and maintenance (${E}_R$). Assimilation, locomotion and behaviour influence the energy uptake of food into the body.

DEB categorizes the metabolic system into four groups: food (X), reserve (E), structure (V) and defecation (P) using the following equation (Fig. 1, Equation (1)):


(1) \begin{align*} \textrm{X}=\textrm{V}+\textrm{E}+\textrm{P} \end{align*}

The energy flux of ingested food ($\dot{P_X}$) is mobilized into the flux of assimilation ($\dot{P_A}$) and defecation ($\dot{P_P}$). The reserve (E) is utilized for somatic allocation (k) and anything else (1 − *k*; e.g. energy allocation towards maturation) in which the energy is further mobilized to somatic maintenance ($\dot{P_S}$) and reproductive maintenance ($\dot{P_R}$) as well as somatic growth ($\dot{P_G}$) and reproductive growth ($\dot{P_R}$) leading to maturation (${E}_H$) and reproductive buffer (${E}_R$) (Fig. 1) (Koojiman, 2009). Importantly, DEB allows one to calculate how much metabolic capacity can be dedicated to a biological process of interest by understanding how other variables may behave and contribute at the individual level, which is particularly useful to estimate population level effects in the rapidly changing climate (Lavaud *et al*., 2021).

For example, based on the DEB model, the metabolic capacity for growth performance prior to the onset of sexual maturation can be expressed as the following (Equation (2)):


(2) \begin{equation*} \dot{P}_G= \dot{P_X} - (\dot{P_A} + \dot{P_P}+ \dot{P_S}) \end{equation*}

where the energy influx of somatic growth ($\dot{P_G}$) is the surplus energy from food energy intake $(\dot{P_X})$ following the energetic costs for digestive processes ($\dot{P_A}$ + $\dot{P_P}$) and somatic maintenance ($\dot{P_S}$). From here, one can estimate the energy fluxes of each variable in which the total energy content (X) can fluctuate with seasonal variability, whereas $\dot{P_X}$ can be strongly influenced by feeding and/or anxiety-related behaviours during predator–prey interactions (Nilsson *et al*., 2012). For instance, studies have reported that fish exposed to OA could increase or decrease their activity (Ferrari *et al.*, 2011; Porteus *et al.*, 2018). While an increase or a decrease in activity may be inferred as a positive adaptive response because of the increased energy intake or energy conservation, any changes in activity can be also interpreted as a negative effect for energy budgets because increased activity will decrease the net energy balance, while a decrease in activity can result in reduced food search activity, leading to a reduction in energy intake ($\dot{P_X}$).

DEB categorizes the life stage into three groups of embryo, juvenile and adult based on the energetic perspectives, but it is important to recognize effects of developmental plasticity on energy metabolism (Schreck and Tort, 2016; Burggren, 2020). During early life stages environmental effects are often additive or cumulative following a logistic S-curve (i.e. Von Bertalanffy function). Under this scenario, it is plausible to expect overall reduced energy flux for growth ($\dot{P_G}$) due to constantly increased somatic maintenance costs ($\dot{P_S}$) under OA. Hence, it is more relevant to evaluate effects of OA on combined early life stages (embryo and juvenile together) to get a holistic understanding of the long-term impacts that OA could have throughout the life history (Moreira *et al.*, 2022). Later in life, fish will prioritize energy for gametogenesis, vitellogenesis and mating behaviours that could have direct impacts on reproduction. A recent study demonstrated that OA could enhance reproduction in orange clownfish (*Amphiprion percula*) (Welch and Munday, 2016). The mechanism behind the improved reproduction is yet to be investigated. However, under the concept of DEB, it is plausible to expect that OA influenced other energy budgets such as locomotion ($\dot{P_X}$) and food assimilation ($\dot{P_A}$ and $\dot{P_P}$) leading to increased fecundity ($\dot{P_R}$).

In the past decade, our understanding on the effects of OA on behaviour and physiology in fish has remarkably improved (Ishimatsu *et al*., 2005; Harvey *et al*., 2013; Jutfelt *et al*., 2013; Heuer and Grosell, 2014; Esbaugh, 2018; Munday *et al*., 2019), featuring recent meta-analyses that reviewed on effects of temperature and OA on metabolic rates (Lefevre, 2016; Cattano *et al*., 2018). In addition, a recent paper showed negative effects on growth and reproduction in marine fish (Hu *et al*., 2024). The OA research field has also experienced a lot of controversy and scrutiny over the publication bias of only positive or negative results, particularly surrounding the effects on olfactory behaviour in fish (Clark *et al*., 2020; Munday *et al*., 2020; Clements *et al*., 2022; Esbaugh, 2023). As a result of this controversy, the research area of OA, perhaps more than any on other abiotic stressors, has been open to the publication of neutral effects, and as such, has become less biased than other research areas of environmental physiology (e.g. hypoxia or temperature). However, what remains poorly understood are the holistic effects of OA on energetic budgets associated with behavioural and physiological changes that could potentially influence their growth and reproduction. In this review, we addressed two following questions: 1) does ocean acidification affect the energy budget of marine fish? and 2) if so, are there differences in how ocean acidification affects energy allocation between life stages, in particular between developing fish and adults? Under the framework of DEB, we used metabolic rate as the proxy for energy metabolism and total energy availability (Fig. 1). We chose locomotion, food assimilation, growth and reproduction as these traits could strongly influence energy dynamics in the DEB model. We combined pre- and post-metamorphic stages into early life stages and differentiated these from adult as we stated above. At the end, we proposed future avenues to improve our understanding of the effects of OA on marine biota, as this approach allows us to take mechanistic approaches and formulate new hypotheses to be tested out in the future, which will facilitate development of strategies for resource management and conservation of biodiversity in the Anthropocene.

## Materials and Methods

From here on we will refer to the replication of ocean acidification in lab studies as simulated ocean acidification (sOA). We searched the literature on Web of Science using the following terms for each category:

Metabolic rate [“ocean acidification” (All Fields) AND fish (Topic) AND “metabolic rate” OR “oxygen consumption rate” (All Fields) AND marine (All Fields) NOT macroinvert^*^ OR invert^*^ (All Fields) and Review Article (Exclude—Document Types) and Associated Data and Book Chapters (Exclude—Document Types) and Editorial Material (Exclude—Document Types)].

Locomotion [“ocean acidification” (All Fields) AND fish (Topic) AND c-start OR “fast start” OR “startle response” OR activity OR swim^*^ (All Fields) AND marine (All Fields) NOT macroinvert^*^ OR invert^*^ (All Fields) and Review Article (Exclude—Document Types) and Associated Data and Book Chapters (Exclude—Document Types) and Editorial Material (Exclude—Document Types)].

Food assimilation [“ocean acidification” (All Fields) AND fish (Topic) AND digestion OR “specific dynamic action” OR SDA OR appetite OR feeding^*^ OR hunger (All Fields) AND marine (All Fields) NOT macroinvert^*^ OR invert^*^ (All Fields) and Review Article (Exclude—Document Types) and Associated Data and Book Chapters (Exclude—Document Types) and Editorial Material (Exclude—Document Types)].

Growth [“ocean acidification” (All Fields) AND fish (Topic) AND growth OR weight OR mass OR lenght (All Fields) AND marine (All Fields) NOT macroinvert^*^ OR invert^*^ (All Fields) and Review Article (Exclude—Document Types) and Associated Data and Book Chapters (Exclude—Document Types) and Editorial Material (Exclude—Document Types)].

Reproduction [“ocean acidification” (All Fields) AND fish (Topic) AND reproduction or fecundity or gonad or gamete^*^ (All Fields) AND marine (all Fields) NOT macroinvert^*^ OR invert^*^ (All Fields) and Review Article (Exclude—Document Types) and Associated Data and Book Chapters (Exclude—Document Types) and Editorial Material (Exclude—Document Types)].

We focussed on empirical studies and excluded any papers on modelling approaches and invertebrates. Where there was more than one environmental variable other than carbon dioxide (e.g. temperature), we only considered the effects of sOA on our traits of interest alone or at the control level of the other variable (i.e. effects of OA at the control temperature). Also, when a study was conducted on multiple species and traits of interest, we chose to count these results separately. It is important to note that where the life stage was not explicitly mentioned, we categorized the life stage to be juvenile in a number of papers based on the size reported. We chose only the literature with an experimental manipulation of PCO_2_ up to 3000 μatm, as this level is not uncommon in coastal areas (Baumann, 2019; Bednaršek *et al*., 2020), but also decided to include some levels that were slightly above this as we considered these studies important in obtaining mechanistic insight into some of the responses being considered. Additionally, we excluded short-term exposures less than 24 hours.

Following the manual curation stated above, we retrieved a total of 202 research articles (39 papers for metabolic rate, 51 papers for locomotion, 11 papers for food assimilation, 87 papers for growth and 14 papers for reproduction) from Web of Science between May and June 2023 (Supplementary Tables). We counted effects of sOA on each trait as positive, negative and no change based on directional changes in energy budgets and then plotted the figures for each measurement.

## Effects of sOA on Metabolic Rate

Metabolic rate is a rate of energy expenditure to maintain organismal integrity and support routine activity, both of which are supported by aerobic metabolism. Thus, metabolic rate provides important insights of how aerobic metabolism is regulated to support biological functions in response to environmental conditions (Clark *et al*., 2013; Chabot *et al*., 2016; Norin and Clark, 2016). Standard metabolic rate (SMR) is the indispensable minimum energy expenditure to sustain life whereas maximum metabolic rate (MMR) is the maximal rate of energy turnover, setting the theoretical ceiling of aerobic metabolism. The difference between SMR and MMR is aerobic scope, which represents the total energy budget or metabolic capacity available for supporting activities such as locomotion, migration, growth and reproduction. Importantly, during early life stages, much of the aerobic metabolism is ontogenetically dedicated to development and growth ($\dot{P_G}$) due to the ecological role of body size in survival whereas reproduction ($\dot{P_R}$) becomes the main component later in life (Sogard, 1997; Miller *et al*., 2009; Palstra and Van Den Thillart, 2010). Theories have speculated that OA could have an allostatic load on aerobic metabolism because additional metabolic costs for acid–base/ion regulation will increase SMR ($\dot{P_S}$) whereas elevated CO_2_ could reduce gas exchange and thus MMR (Pörtner and Farrell, 2008).

**Figure 2:**
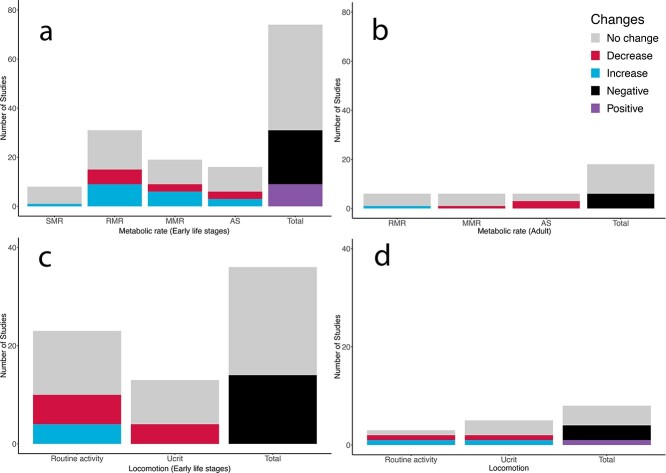
Number of studies that examined the effects of simulated ocean acidification (sOA) on metabolism of early life stages (a), metabolism of adults (b), locomotion of early life stages (c) and locomotion of adults (d). For individual figures we noted the number of studies reporting significant increases (blue), decreases (red) or no effect (grey) of sOA in the studies summarized. However, sometimes any change could be considered negative, as both an increase or a decrease could be negative such as changes in routine activity. Therefore, in the total columns the number of studies showing a negative effect (black), a positive effect (purple) and no effect (grey) are summarized in order to determine the overall effect of sOA on that measure.

In contrast to theoretical predictions, our results demonstrate there seems to be no clear evidence for an effect of sOA on metabolic rate of early life stages and adult stages, which agree with previous reviews on the topic (Lefevre, 2016; Cattano *et al*., 2018). In early life stages, we found 37 research articles measuring at least one metric for metabolic rate (i.e. SMR, RMR, MMR or AS), and that effects of sOA on metabolic rate were equivocal in early life stages with nine studies of positive effect, 22 studies of negative effects and 43 studies of no significant change (Fig. 2a). In adult fish, the effects of sOA were also equivocal, with no study reporting positive effect, six studies reporting negative effects and twelve studies showing no significant change (Fig. 2b).

### Standard metabolic rate

SMR is the minimum energy expenditure to sustain life, which taxes the total energy available in the DEB model. Our analysis showed only one study reporting a significant increase while the other seven studies did not find a difference between treatments (Fig. 2a). Although we did not find any studies on adult fish, it is plausible to expect that effects should be minimal due to their physiological robustness (Brauner *et al*., 2019). These findings suggest that effects of sOA on metabolic costs for maintenance may be negligible on both early life stages and adult of marine fish, which agrees with a recent review (Alter *et al*., 2024). Indeed, the contribution of acid–base regulation and ion transport to SMR in fishes is thought to be less than 5%, and also the acid–base compensation process is known to be very rapid (Hochachka and Somero, 2001; Michaelidis *et al*., 2007; Brauner *et al*., 2019). Although some fish species may be more metabolically sensitive to sOA, our findings suggest that OA may have marginal effects on somatic maintenance ($\dot{P_S}$) in terms of acid–base/ion regulation in both early life stages and likely also in adults.

### Routine metabolic rate

RMR is a useful physiological metric to approximate energetic expenses for the daily activity, which can strongly influence the energy intake ($\dot{P_X}$). In early life stage, we have found 31 research articles with nearly half of them showing either an increase (9) or a decrease (6) and over half showing no significant change (16) (Fig. 2a). Whereas in adult fish, we found one study showing an increase and five studies reporting no change (Fig. 2b). Although sOA may have minimal effects on maintenance costs ($\dot{P_S}$; SMR) in both life stages, our RMR results implied that sOA could reduce the energy uptake from food ($\dot{P_X}$) due to increased energy expenditure towards locomotion or decreased foraging activity. This has important implications because it subsequently reduces the net energy balance for development and growth ($\dot{P_G}$) in early life stages as well as for reproduction ($\dot{P_R}$) in adults.

### Maximum metabolic rate

MMR represents the maximum aerobic metabolism closely associated with the cardiorespiratory capacity of an organism (Hillman *et al*., 2013). Thus, MMR pinpoints the theoretical ceiling of aerobic metabolism, which enables to calculate aerobic scope (the total energy budget available for $\dot{P_G}$ and $\dot{P_R}$). In early life stage, we have found a total of 19 studies with almost half of them showing either an increase (6) or decrease (3) and the rest showing no significant change (10) (Fig. 2a). In adults, we found only one study reporting a significant decrease and five studies reporting no significant changes in MMR (Fig. 2b).

While a reduction in MMR is related to cardiorespiratory responses to elevated CO_2_ (Heuer and Grosell, 2014; Lefevre, 2016), it is interesting to note that mild CO_2_ levels may increase MMR and potentially the energy budget in some fish. It has been hypothesized that an exposure to elevated CO_2_ may increase MMR via a Root effect mediated oxygen delivery mechanism in aerobic muscle in fishes (Rummer *et al*., 2013); however, our data suggest that, at best, this would only apply to a subset of fish species (Lefevre, 2016). As such, we also concluded that sOA does not affect MMR in either early life or adult stages.

### Aerobic scope

AS represents the aerobic capacity that can be dedicated towards biological functions above basal maintenance, which represents the physiological capacity for development, growth and reproduction (Fry, 1957). In early life stages, we have found a total of 16 research articles with less than half of them showing either an increase (3) or a decrease (3) and over half of them no significant changes (10) (Fig. 2a); whereas in adults, we found three studies reporting an increased AS and three other studies with no significant change (Fig. 2b). Our results suggest that OA may have only a marginal effect on the total energy budget on both early life stages and adults.

## Effects of sOA on Locomotion

In our review, we chose routine activity and critical swimming speed within the context of energy budget because from an ecological fitness perspective, changes in these traits could significantly influence energy uptake from food ($\dot{P_X}$) and the net energy balance for growth or reproduction (E) (Kikkawa *et al*., 2003; Munday *et al*., 2008; Bignami *et al*., 2017). In early life stages, we found a trend of negative effects of sOA on locomotion with no studies showing positive effects, 20 studies showing negative effects (4 for increase and 16 for decrease) and 24 studies reporting no significant effect (Fig. 2c). In adult fish, only one study found a positive change and five studies demonstrated a negative effect while the remaining five studies showed no change in locomotion in response to sOA (Fig. 2d). Overall, the equivocal results of sOA on locomotion during early life stages may be related to heightened activity masking the physiological effect. In the following sections, we consider the effects on routine activity and critical swim speed separately as both have the potential to affect the energy allocation in different ways.

### Routine activity

Routine activity has direct impacts on energy budgets as activity can make up to 40% of energy expenditure (Boisclair and Leggett, 1989). We found that almost the half of studies reported the negative effect of sOA on routine activity in early life stages although the effects seem to be more pronounced in adult (Fig. 2c and d). While the negative effects of reduction in locomotion may be the result of a physiological acclimatization in response to sOA, these results implied that sOA could decrease the net energy balance for growth and reproduction (Munday *et al*., 2019). Although a more pronounced effect of sOA on locomotion in adult could be due to a smaller sample size, it is possible that the smaller effect on early life stages could reflect relatively higher activity, which could have masked the effects of sOA. It has been proposed that changes in activity in response to OA could be due to heightened anxiety or reduced olfactory responses (Nilsson *et al*., 2012; Porteus *et al*., 2018). However, we found that a lot of studies often lack physiological mechanisms associated with changes in activity. As activity is directly related to energy and muscle metabolism, additional studies with a focus on muscle metabolism such as muscle enzyme activity and histology could provide more insights for explaining these variable results in the literature.

### Critical swimming speed

Critical swimming speed (Ucrit) is measured by incrementally increasing water velocity until a speed at which fish cannot maintain the normal swimming behaviour (Plaut, 2001). It is worth to note that Ucrit does not necessarily translate into the maximum aerobic swimming speed as it is well documented that anaerobic metabolic activity starts occurring at 60–80% of Ucrit (Wilson and Egginton, 1994; Lee *et al*., 2003; Zhang *et al*., 2019). Although Ucrit may not be directly related to the energy budget from a conventional perspective, it is an important physiological metric to represent the locomotory performance for migration and foraging that influences catching prey and thus energy uptake ($\dot{P_X}$). Intuitively, it is thought that newly hatched larvae may have a limited swimming capacity and passively drift along the current. However, the swimming speed of 50 coral reef fish species in their larvae stage demonstrated a high mean Ucrit of 20.6 cm/s (Leis and Carson-Ewart, 1997), and therefore, Ucrit is also an important indicator of locomotory performance in larvae as well.

There were 13 studies measured Ucrit in fish during early life stages, with the majority focused on coral reef fish species in which four studies showed a decrease in Ucrit whereas nine studies showed no significant difference between sOA and control groups (Fig. 2c). In adult, there were only two papers on five fish species in which one species showed a significant increase; in another study, one showed a significant decrease and the other three showed no change in Ucrit (Fig. 2d). It appears that sOA does not affect Ucrit in either life stage, but may increase Ucrit in some adult fish, possibly due to enhanced oxygen uptake as we discussed above.

**Figure 3:**
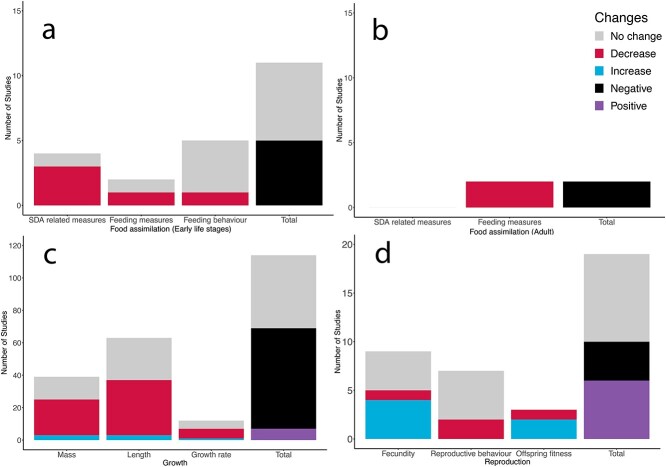
Number of studies that examined the effects of simulated ocean acidification (sOA) on food assimilation of early life stages (a), food assimilation of adults (b), growth of early life stages (c) and reproduction of adults (d). For individual figures, we noted the number of studies reporting significant increases (blue), decreases (red) or no effect (grey) of sOA in the studies summarized. However, sometimes any change could be considered negative, as both an increase or a decrease could be negative, such as changes in routine activity. Therefore, in the total columns the number of studies showing a negative effect (black), a positive effect (purple) and no effect (grey) are summarized in order to determine the overall effect of sOA on that measure.

## Effects of sOA on Food Assimilation

Food assimilation ($\dot{P_A}$) is crucial in the DEB as it taxes the total energy input ($\dot{P_X}$), which subsequently gives rise to somatic growth ($\dot{P_G}$) and reproduction ($\dot{P_R}$). Specific dynamic action is the sum of metabolic costs of digesting a meal including ingestion, digestion, absorption, assimilation and egestion (SDA; $\dot{P_A}$+ $\dot{P_P}$), which can reach near or even exceed MMR in some fishes (Steell *et al*., 2019). For early life stages, we found that only nine studies have looked at the effects of sOA on food assimilation of developing fish (Fig. 3a), including only one study that directly measured the effects of sOA on SDA. In adults, we found only two studies assessed the effect of high PCO_2_ on digestion and/or feeding behaviour in adult marine fish, and all show negative effects of sOA on food assimilation measures (Fig. 3b). While acknowledging the limited number of research articles, OA seems to have a negative effect on digestion and feeding activity of both early life stages and adult marine fish. This can result in a reduction in the net energy balance ($\mathrm{E}$) under high CO_2_, which can significantly impact on fish growth (see below). Thus, more studies are necessary to investigate the changes in digestive physiology under OA.

## Effects of sOA on Growth

**Figure 4:**
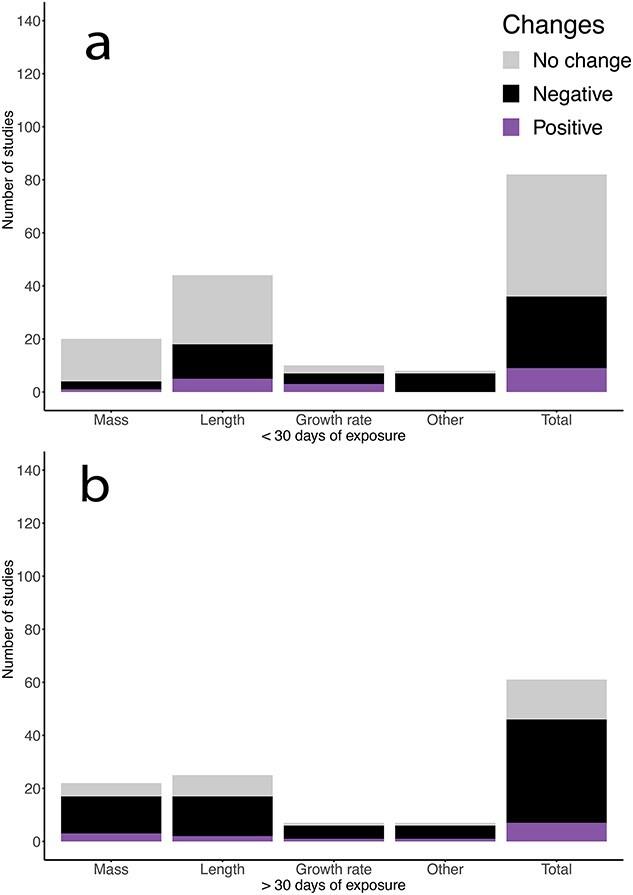
Number of studies that examined the effects of simulated ocean acidification (sOA) on growth of fish by exposure durations of (a) less than 30 days and (b) 30 days or more. The number of studies showing a negative effect (black), a positive effect (purple) and no effect (grey) are summarized in order to determine the overall effect of exposure time to sOA on different growth measures.

During early life history, energy metabolism is ontogenetically prioritized for growth and development (k) due to its role in survival and fitness (Arendt, 1997; Sogard, 1997). Our literature search has illustrated an overall negative effect on growth (Fig. 3c), which agrees with a recent meta-analysis paper (Alter *et al.,* 2024). Out of 87 papers, more than half reported a negative effect of sOA on growth-related measures such as body mass, length or growth rate, while ~ 40% reported no effect and a very small percent (~6%) reported a positive effect (Fig. 3c). It is plausible that during shorter exposure times, growth could be affected by a transient effect of sOA on ion and acid–base regulation, whereas longer exposures could result in additive and/or cumulative effects on energy budgets. Therefore, we decided to divide papers by time of exposure either longer or shorter than 30 days of exposure to understand longer and shorter-term impacts of sOA on growth (Fig. 4). When only looking at longer term exposure studies, there were fewer studies showing no effect and a larger proportion of studies showing a negative effect on growth measures. This indicates that in many species, the longer fish are exposed to sOA, the more likely they are to be negatively impacted by sOA, perhaps due to a cumulative effect on energy budgets and thus energy allocation towards growth ($\dot{P_G}$). These findings are consistent with larval cobia (*Rachycentron canadum*) showing a reduced time to starvation after exposure to 1700–2100 μatm for 5–13 days (Bignami *et al.*, 2017). While starvation resistance is not a direct measure of growth, it reveals catabolic responses of energy reserves such as structure (V) and storage (E), which are strongly influenced by growth performance in young fish. The mechanism of changes in anabolism/catabolism under OA needs to be further investigated, but we hypothesize that reduced growth performance caused by sOA may be more closely related to the efficiency of food assimilation ($\dot{P_A}$). Importantly, most sOA studies fed fish *ad libitum* during the experimentation whereas food amount is most likely limited in the wild, which may translate a more negative effect on the net energy balance for growth of fish in the wild. More research needs to focus on understanding the longer-term impact of sOA and design studies appropriately as the metabolic impacts of acidification seem to have a small but additive effect on the growth of developing fish.

## Effects of sOA on Reproduction

The energy budget for reproduction (1-k) is the sum of surplus energy after the somatic processes (k). Therefore, changes in any variables in the DEB could have strong influences on reproduction (Kooijman, 2009). Within the reproductive energy budget, reproductive success is dependent upon how much energy can be dedicated to reproductive reserves ($\dot{P_R}$). A total of 14 studies have assessed the effects of sOA on reproduction in marine fish (all on adults). Interestingly, about half the studies reported positive effects on fecundity and related measures (Fig. 3d), including clutch size, number of clutches produced, average egg size, and fertilization success (Miller *et al*., 2013; Welch and Munday, 2016; Faria *et al*., 2018; Petit-Marty *et al*., 2021). OA can be considered a mild stressor for adult fishes due to their ability to quickly compensate through acid–base regulation in as little as 40 minutes (Montgomery *et al*., 2022). Therefore, it is possible that the positive effects of sOA on reproduction could be attributed to hormesis, where the level of the stressor gives rise to opposite responses depending on its severity (Schreck, 2010). This stimulating effect is not well understood, but could happen through the induction of heat shock proteins, which are molecular chaperones that play numerous important roles including stabilizing protein structure as well as energy metabolism (Schreck, 2010). Other studies reported a negative effect (Welch and Munday, 2016) or minimal/no effect of exposure to sOA on reproduction (Miller *et al*., 2015; Lopes *et al*., 2020; Concannon *et al*., 2021). Sometimes these effects were associated with either an increase or a decrease in offspring fitness such as size of larvae at hatch, yolk size at hatch, survival or body size a month post hatch (Miller *et al*., 2013; Welch and Munday, 2016; Faria *et al*., 2018), indicating that there could be tradeoffs between fecundity and quality of offspring. However, this is not uncommon in highly fecund species such as fish that favours maternal provision over offspring size (Einum and Fleming, 2000). Moreover, an exposure to sOA may have negative effects on the reproductive behaviours of males such as time spent carrying parental behaviours, mating, nest defense as well as sneaker male behaviour as previously shown (Fig. 3d) (Milazzo *et al*., 2016; Spatafora *et al*., 2021); however, two studies did not find an effect of sOA on reproductive behaviours including one conducted at CO_2_ seeps (Milazzo *et al*., 2016; Sundin *et al*., 2017). Overall, it seems that sOA has the potential to improve fecundity in some fish species with favouring energy allocation to reproductive development ($\dot{P_R}$), but this comes with a tradeoff of decreased male reproductive behaviours or offspring fitness.

## Effects of sOA on Energy Budgets

**Figure 5:**
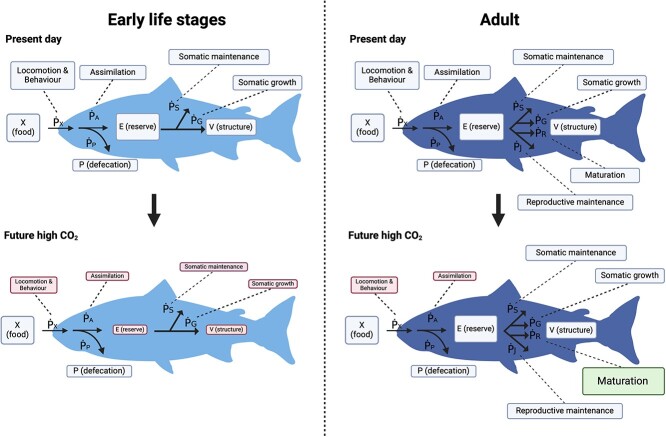
Predicted effects of simulated ocean acidification (sOA) on energy budgets in early life stages (left) and adult (right) fish. The red boxes represent functions that could be negatively affected by ocean acidification in the future and green boxes represent functions that could be positively affected in the future. For both adults and developing fish these are related to food assimilation, feeding related behaviours and locomotion. In early life stages we predict negative effects on somatic maintenance and growth. In adults, there are some potential positive effects of sOA on maternal reproductive fitness, perhaps at the cost of locomotion or food assimilation.

OA will likely have negligible effects on aerobic metabolism in both early life stages and adult as concluded by other recent meta-analysis papers (Lefevre, 2016; Cattano *et al*., 2018), but OA could negatively affect activity, digestion and growth on early life stages as revealed by our synthesis (Fig. 5). This indicates that there will not be substantial changes in the total energy throughout the life history, but an evident shift in the energy balance in early life stages, and to a lesser extent in adults in response to OA (Fig. 5).

We also noted that longer-term studies tend to report a negative effect of sOA on growth in agreement with a recent meta-analysis (Cattano *et al*., 2018), suggesting that there are small, but additive effects of OA evident during early life stages. The reduced growth could be due to negative effects of OA on the decreased food intake ($\dot{P_X}$) and/or inefficient digestion ($\dot{P_A}$) in some species, which could give rise to reduced energy availability for growth ($\dot{P_G}$). This could result in more time spent at a vulnerable life stage and thus become more prone to predation risk (Wang *et al*., 2017; Domenici and Seebacher, 2020). Importantly, most studies to date have been conducted under *ad libitum* food conditions, which is not a realistic scenario for wild fish; therefore, our results may translate into more time spent foraging and thus reduce the net energy balance for maintenance in developing fish (Stiasny *et al*., 2018). Although many previous studies have discussed the behavioural or physiological effects of OA under the concept of energy metabolism and implications for ecological fitness (Stiasny *et al*., 2018; Schwieterman *et al*., 2019), no study to date has measured all of these responses related to energy budgets within one single study or species. Therefore, future research should take a more holistic approach understanding the physiological mechanism related to energy budgets under OA so that we can better determine the full impacts of OA on developing fish.

Additionally, in adults, sOA might have some positive effects on fecundity possibly due to hormesis, but at a potential cost to reduced offspring fitness. Collectively, the effects of sOA on adult and offspring might translate into a strong effect of sOA at a population level, again consistent with previous findings (Cattano *et al*., 2018). Taken together, DEB would predict that in some species OA could reduce food intake ($\dot{P_X}$), efficiency for digestion ($\dot{P_A}$), which would result in overall decreased energy reserves (E) during early life stages (Fig. 5). In adults, OA could lead to an increase in reproduction ($\dot{P_R}$), but it may come with selective decreased energy budgets for locomotion ($\dot{P_X}$) and food assimilation ($\dot{P_A}$) (Fig. 5).

Although excluded from our formal investigation, temperature alone seemed to have larger effects than OA. It is important to note that fish would not experience OA alone in the wild, but in combination with other environmental stressors such as elevated temperature and hypoxia (Tigert and Porteus, 2023). As temperature effects can be additive or synergistic (Schulte, 2011), more research needs to be conducted to understand how OA interacts with other stressors to influence energy budgets in the context of ongoing climate change (Hu *et al*., 2024).

The most striking observation was the limited number of studies that have been conducted on adult fish. This could be perhaps due to their better capability to deal with environmental perturbations than young fish (Brauner *et al*., 2019) or due to logistical constraints associated with the ease of working with smaller fish. Therefore, we strongly recommend that future research should take a holistic approach of the DEB to reveal mechanistic insights for metabolic shifts of adult fishes in response to OA so that we can better inform management strategies for conservation of vulnerable species and to address biodiversity in the near future.

## Supplementary Material

Web_Material_coae069

## Data Availability

Data generated for figures are available as supplementary data on the online version.
